# Microbial load and safety of paper currencies from some food vendors in Jimma Town, Southwest Ethiopia

**DOI:** 10.1186/1756-0500-7-843

**Published:** 2014-11-25

**Authors:** Gosa Girma, Tsige Ketema, Ketema Bacha

**Affiliations:** Department of Biology, Jimma University, College of Natural Sciences, Jimma, Ethiopia

**Keywords:** Ethiopia, Food vendors, Jimma, Microbial load, Microbial safety, Paper currency

## Abstract

**Background:**

Paper currency is used for every type of commerce and plays an important role in the life of human beings. However, the combination of its widespread use and constant exchange make paper currency a likely agent for disease transmission. Thus, the aim of this study was to evaluate the microbial load and safety of Ethiopian paper currencies collected from some food vendors in Jimma town.

**Methods:**

Standard microbiological methods were used for the enumeration of various microbial groups, isolation and characterization of pathogenic bacteria and their growth potential in selected weaning foods. A total of 100 samples of Ethiopian paper currencies, consisting of five denominations, from street food venders, hotels and cafeterias in Jimma town were collected aseptically. Sterile cotton swabs moistened with buffered peptone water solution were used for swabbing and the swabs were separately soaked into 10 ml sterile buffered peptone water solution.

**Results:**

Mean microbial counts of Aerobic mesophilic bacteria, Staphylococci, Enterobacteriaceae, coliforms and Aerobic bacterial spores were (log CFU/cm^2^) 6.32, 4.43, 3.14, 2.98 and 3.78, respectively. However, mean counts of Yeasts and Moulds were below detectable levels. There was statistically significant variation (p < 0.05) among the mean counts of microbes isolated from samples of paper currencies. The predominantly isolated microbial groups were *Staphylococcus* spp. (34.06%) followed by *Bacillus* spp. (31.88%), Enterobacteraceae (13.39%), *Micrococcus* spp. (9.55%) and *Streptococcus* spp. (9.03%). Overall, 25% and 10% of the samples were positive for *S. aureus* and *Salmonella* spp, respectively. In challenge study, *Salmonella* spp. and *S. aureus* reached the infective dose within 12 to 18 hours of inoculation.

**Conclusion:**

Thus, paper currencies could be considered as one of the possible vehicles for transmission of disease causing microorganisms. Poor handling practices and personal hygiene of the food vendors could contribute to the observed microbial counts. Thus, it calls for awareness development on the potential risks associated with poor handling of paper currencies at all level of the food establishments.

## Background

Trade has been practiced by human from time immemorial and money is an indispensable part of it since its introduction in China approximately 1000 AD [1]. We use money as a measuring unit in pricing a transaction, offer it as a medium for exchange of goods and services, settlement of debts, for deferred payments in economic activities and make it a store of value for our savings. When used as medium of exchange, paper currencies could be handled under unhygienic conditions and possibly contaminated with different microbes [2, 3], making it a prime multiplication medium for various microorganisms and could constitute a major health hazard [4].

Many people do not care to the level of cleanness of their fingers when handling money and pick paper currencies with contaminated hands, leading to the contamination of paper currency notes with microorganisms [5]. Furthermore, market men and women squeeze paper currencies and put them into their dirty pockets. Meat sellers in slaughter houses and at market places usually collect money from buyers with hands contaminated with blood and animal wastes [5, 6]. Such money handling habits can introduce microbes to the notes. In most parts of the world, it is believed that the simultaneous handling of food and money/currency/ contributes to the incidence of food-related public health incidents [7]. Data accumulated during the last 20 years indicate that pathogens on currency notes could represent a potential cause of foodborne illness [8]. Evidently, many food outlets rely heavily on paper currencies for exchange with high frequency of contact between the currencies and foods [9] risking the safety of consumers [10].

According to reports made from several studies, many bacterial groups such as *Citrobacter* sp., *Mycobacterium lepirae*, *Salmonella* sp., *Escherichia coli, Staphylococcus aureus, Klebsiella* sp, *Streptococcus* spp*.* and *Pseudomonas aeroginosa* were found associated with paper currency notes [3, 11]. Paper currencies were also reported contaminated with fungi including *Aspergillus niger, A. flavus, A. paraziticus, Candida* spp. *Penicillium* spp. and *Rhizopus* spp.*, Alternaria tenuis, Trichoderma* spp., *Fusarium* spp., and *Sporotrichum* spp. [3, 8, 11–14].

Microbial contaminants of paper currencies could be from several sources including counting machine, atmosphere, storage environment, usage, handling or production [15, 16]. Moreover, the contamination of currency notes could be traced to dust, soil, water, body of currency handlers (such as hand, skin, and wounds). Furthermore, many people tongue-wet their fingers when counting money thereby, contaminating their fingers as well as currency notes [2].

Though several studies reported on the level of microbial contamination of paper currencies from different parts of the world including Africa, to-date to the authors’ knowledge there was no report on its status from Ethiopia. Therefore, the purpose of this study was to evaluate the microbial load and safety of Ethiopian paper currencies collected from street food vendors, hotels and cafeterias in Jimma town, southwest Ethiopia.

## Methods

### Sample collection

The study was conducted in Jimma town which is located at 353 km southwest of Addis Ababa, the capital city of Ethiopia. A total of hundred (100) paper currency samples currently in circulation involving five denominations (1, 5, 10, 50 and 100), 20 notes each, were randomly collected from street food venders, workers of hotels and cafeterias in Jimma town. In addition, 20 newly minted paper currencies, obtained from Commercial Bank of Ethiopia, Jimma Branch were used as negative control. The samples were collected aseptically by letting the individuals to drop the paper currencies into a sterile polythene bags. The polythene bags were promptly sealed and the individuals were given a replacement equivalent to what they deposited in the polythene bags. The polythene bags were immediately transported to Jimma University, Research and Postgraduate Studies Laboratory for microbial analysis. Thereafter, a sterile cotton swab moistened with buffered peptone water solution was used for swabbing thoroughly on both surfaces of each sampled paper currency placed on pre-sterilized aluminum foil that was larger than the size of paper currencies and the swabs were separately soaked into 10 ml sterile buffered peptone water solution. The samples were kept in refrigerator at 4°C until microbial analysis was conducted within one to two hours.

### Microbiological methods

The collected paper currency samples were analyzed for microbial load and safety. Accordingly, 1 ml of each paper currency swab sample was transferred aseptically into 9 ml of buffered peptone water (BPW) and thoroughly mixed using vortex. The homogenates were serially diluted from 10^-1^ to 10^-6^ and a volume of 0.1 ml aliquot of appropriate dilution was spread-plated in duplicate on pre-solidified plates of Plate Count Agar (Oxoid), MacConkey Agar (Oxoid), Violet Red Bile Agar (Oxoid), Mannitol Salt Agar (Oxoid) and Potato Dextrose Agar (Oxoid) plates supplemented with 0.1 g chloramphenicol and incubated at optimum temperature and time for counts of aerobic mesophilic bacteria and aerobic bacterial spore, Enterobacteriaceae, coliforms, staphylococci, yeasts and moulds, respectively. Plates with colonies between 30 and 300 were considered for counting. Both lactose fermenting pink to purple mucoid colonies and non-lactose fermenting colorless colonies on MacConkey agar were enumerated as member of Enterobacteriaceae. Purplish red colonies surrounded by reddish zone of precipitated bile on VRBA agar were counted as coliforms while formations of golden yellow colonies on mannitol salt agar were counted as staphylococci. Smooth (non-hairy) colonies without extension at periphery were counted as yeasts whereas hairy colonies with extension at periphery were counted as moulds. The counted colonies (mean values of duplicate plates) were expressed in colony forming units per square centimeter (CFU/cm^2^) of the sampled paper currencies and then converted into log colony forming units (log CFU/ cm^2^) for data management.

### Microbial analysis

After enumeration of aerobic mesophilic bacteria, 10 to 15 colonies with distinct morphological differences like color, size and shape were randomly picked from countable plates and aseptically transferred into tubes containing 5 ml Nutrient Broth (Oxoid)*.* These were incubated at 30-35°C over night. Cultures were purified by repeated plating and preserved on Nutrient agar slants at 4°C until characterization was carried out. Then isolates were characterized to the genus level and various bacterial groups according to John [17] bacterial identification method. Accordingly, isolates were characterized for cell morphology (cell shape, cell grouping), motility, Gram reaction, O/F test (Oxidative or fermentative utilization of glucose), catalase test and cytochrome oxidase test. Furthermore, for identification of presumptive *Staphylococcus aureus* and *Salmonella* spp., the distinct colonies were purified and characterized using standard microbiological methods [18, 19]. Accordingly, Gram-positive cocci with clustered arrangement under microscope were considered staphylococci and subjected to further biochemical tests (including catalase, cytochrome oxidase, and coagulase tests). Catalase test was carried out by flooding young colonies with a 3% solution of hydrogen peroxide (H_2_O_2_). The formations of bubbles were considered as the presence of catalase. Furtermore, cytochrome oxidase test was conducted using the method out lined by Kovacs [20]. Briefly, freshly prepared 1% Î± naphthalene in absolute ethanol and 1% N, N, – dimethyl –p- phenylenediammonium chloride in distilled water were mixed in the ratio of 2:3 just before use. After flooding the young colonies with the mixture on Nutrient Agar plates, appearance of a blue color on the colonies within 30 seconds to 2 minutes were considered as an oxidase positive reaction and negative when color is not changed. For identification of *S. aureus,* slide coagulase test was done. In slide test, which detects bound coagulase, colonies of the purified isolates were emulsified in a drop of distilled water on two separate slides to make thick suspensions. A loopful of plasma was added to one of the suspensions and mixed gently. Observation of the clumping within 5 -10 seconds was considered that the organism was coagulase producer. In general, isolates that were Gram-positive, coccoid, catalase positive and capable of coagulating human plasma were considered as *S. aureus.*

For isolation of *Salmonella* spp., cotton swab sample of each paper currency was aseptically transferred into test tube containing 10 ml of buffer peptone water (BPW), homogenized for 5 minutes and then incubated at 37°C for 24 hours (for recovery and proliferation of cells which might be injured during processing in addition to making the number of target organisms grow to a detectable level). Following the primary enrichment in buffer peptone water, 1 ml of the culture was transferred onto 10 ml of Selenite cysteine broth (Oxoid) and incubated at 43°C for 48 hours (to inhibit the growth of none targeted microorganisms like gram positive bacteria and Coliforms besides further enrichment of *Salmonella spp*.). A loopful of culture from Selenite cysteine broth was streaked onto Xylose Lysine Deoxycholate (XLD) agar (Oxoid) and incubated at 37°C for 18 hours. Characteristic colonies were picked, further purified and tested biochemically using gallery of biochemical media (all Oxoid) including Triple Sugar Iron Agar (TSIA), Lysine Iron Agar (LIA), Urea Agar (UA), Simmon’s Citrate Agar (SCA) and Sulfide Indole Motility (SIM) medium [21].

### Growth potential of *Staphylococcus aureus*and *Salmonella*spp. isolated from paper currencies on selected weaning foods

The growth potential of *Staphylococcus aureus and Salmonella* spp. was assessed on selected weaning foods (cow milk and mango juice). Selection of the weaning foods was based on the frequency of use of the same products in the study area, especially by out-patients and guardians visiting the referral hospital in the town. Two hundred (200) ml of each food items was separately homogenized using vortex and steamed at 80°C in water bath for 10 minutes to kill any vegetative cells that might be present in the food items. About 100 ml of each food items was challenged separately with 1 ml overnight cultures of the test strains to bring the final inoculums size to the level of 10^2^-10^3^ CFU ml^-1^. The challenged food was incubated at 30-32°C for 24 hours. To determine the initial inoculum level, 10 ml of each inoculated food was homogenized in 90 ml of BPW and 0.1 ml of appropriate dilution was spread plated on XLD for *Salmonella* spp*.* and MSA for *S. aureus*. A portion of food sample was further sampled aseptically at 6 hours interval from 0-24 hours [22]. The pH of each food sample was measured using digital pH meter from 0 to 24 hours at an interval of 6 hours while assessing the growth potential of the potentially pathogenic test stains.

### Statistical methods

Data was analyzed using SPSS software version 16. All values were expressed as mean ± standard deviation and the mean values of paper currency samples from different sources were compared using one way ANOVA and the significance of differences was considered at 95% confidence interval (*p* <0.05).

## Results

The mean counts of aerobic mesophilic bacteria (AMB), staphylococci and Enterobacteriaceae were the highest in paper currency samples of denomination ten (7.68, 6.32, and 5 log CFU/cm^2^, respectively) followed by denomination one (7.65, 6.03 and 4.13 log CFU/cm^2^), respectively, (Table 1). The lowest mean count was encountered in samples from denomination hundred with the least mean counts of AMB (4.66 log CFU/cm^2^) and below detectable level for all the rest except for aerobic spore former (2.28 log CFU/cm^2^). Likewise, the mean counts (log CFU/cm^2^) of coliforms were the highest in paper currency denomination one (4.41) (Table 1) followed by denomination ten (4.09). But, mean counts of yeasts and molds in all paper currency samples were below detectable levels (<2 log CFU/cm^2^) even on plates inoculated from the original suspension (Table 1). Generally, the mean counts of all bacterial groups were above detectable level (≥2 log cfu/m^2^) in all paper currency samples although the mean counts of Staphylococci (denominator 100) and Enterobacteriaceae and coliforms (denominators 50 and 100) were below detectable levels (Table 1). The maximum mean bacterial count encountered among the paper currency samples was 7.68 ± 0.59 CFU/cm^2^ for AMB (in denomination five). There were statistically significant differences among the mean counts of AMB, staphylococci, Enterobacteriacea, coliforms, aerobic spore counts and yeast of the different denominations except for yeast counts (Table 1). The twenty newly mint currency notes used as least negative controls did not reveal any micro-organism.

**Table 1:** **Mean microbial counts (log CFU/cm** ^**2**^ **) of microbial groups isolated from paper currencies in Jimma town, southwest Ethiopia**

PCD	Sample size	AMB	Staph*.*	Enterob.	Coliforms	ABS	Yeasts	Molds
		Mean ± SD	Mean ± SD	Mean ± SD	Mean ± SD	Mean ± SD	Mean ± SD	Mean ± SD
1	20	7.65 ± 0.53^a^	6.03 ± 3.14^c^	4.13 ± 3.47^e^	4.41 ± 3.00^g^	5.06 ± 3.45^i^	0.89 ± 1.24^k^	0.51 ± 1.05^m^
5	20	6.83 ± 0.96^a^	4.90 ± 3.72^c^	3.25 ± 2.38^e^	3.26 ± 2.35^g^	3.65 ± 2.77^i^	0.25 ± 0.78^k^	0.25 ± 0.76^m^
10	20	7.68 ± 0.59^b^	6.31 ± 3.26^c^	5.00 ± 3.37^e^	4.09 ± 3.44^g^	5.24 ± 3.17^i^	0.63 ± 1.12^l^	0.38 ± 0.93^m^
50	20	4.72 ± 0.11^a^	2.78 ± 2.11^d^	1.69 ± 1.37^f^	1.64 ± 1.29^h^	2.69 ± 2.19^j^	0.26 ± 0.77^k^	0.12 ± 0.55^m^
100	20	4.66 ± 0.07^a^	1.83 ± 1.19^d^	1.64 ± 1.30^f^	1.52 ± 1.15^h^	2.28 ± 2.08^j^	0.25 ± 0.76^k^	0.13 ± 0.56^m^
Total	100	6.32 ± 0.45	4.43 ± 2.68	3.14 ± 2.25	2.98 ± 2.25	3.78 ± 2.73	0.46 ± 0.94	0.28 ± 0.77

Where: PCD = Paper Currency Denominations, AMB = Aerobic Mesophilic Bacteria, Staph. = Staphylococci, Enterob. = Enterobacteriaceae, ABS = Aerobic Bacterial Spore, SD = Standard deviation. Similar superscripts within the same column indicate absence statistically significant differences among the mean values. Mean with different superscript is statistically significant from the others.

### Microbial analysis of paper currencies

From a total of 100 different paper currency samples analyzed for the microbiological safety, a total of 222, 204, 228, 174 and 135 bacterial colony were isolated from among paper currency denomination 1, 5, 10, 50 and 100, respectively (Table 2). Overall, 963 bacterial isolates were obtained, characterized and grouped into a family and six genera. Out of 963 isolates, 814 (84.53%) were Gram positive and 149 (15.47%) were Gram negative bacteria (Table 2). The aerobic mesophilic bacterial count were dominated by *Staphylococcus* spp. 328 (34.06%) followed by *Bacillus* spp. 307 (31.88%) and Enterobacteraceae 129 (13.39%) (Table 2). *Micrococcus* spp. 92 (9.55%), *Streptococcus spp.* 87 (9.03%), *Acinetobacter* spp. 14(1.45%) and *Pseudomonas* spp. 6(0.62%) were also among the aerobic mesophilic bacteria encountered on paper currency samples.

**Table 2:** **Frequency distribution (%) of dominant bacteria isolated from paper currencies collected from food vendors, Jimma town, Southwest Ethiopia**

PCD	No of PCD	No of isolates	*Staphylococcus*spp.	*Bacillus*spp.	Enterobacteriaceae	*Micrococcus*spp.	*Streptococcus*sp.	*Acinetobacter*spp.	*Pseudomonas*spp.
		No	Frequency (%)	Frequency (%)	Frequency (%)	Frequency (%)	Frequency (%)	Frequency (%)	Frequency (%)
1	20	222	71 (31.98)	70 (31.53)	25 (11.26)	26 (11.71)	22 (9.90)	5 (2.25)	3 (1.35)
5	20	204	67 (32.84)	60 (29.41)	40 (19.61)	14 (6.86)	20 (9.80)	3 (1.47)	-
10	20	228	72 (31.58)	79 (34.65)	29 (12.72)	21 (9.21)	20 (8.77)	4 (1.75)	3 (1.32)
50	20	174	62 (35.63)	57 (32.76)	20 (11.49)	18 (10.34)	15 (8.62)	2 (1.15)	-
100	20	135	56 (42.24)	41 (29.31)	15 (11.21)	13 (9.48)	10 (7.76)	-	-
Total	100	963	328 (34.06)	307 (31.88)	129 (13.39)	92 (9.55)	87 (9.03)	14 (1.45)	6 (0.62)

Where; PCD = Paper Currency Denominations. Numbers in parenthesis indicate percentage.

### Prevalence of *Staphylococcus aureus*and *Salmonella*spp

In the present study, of the total 100 paper currency samples, 25% were positive for *S. aureus* (Table 3). It was as prevalent as 10% in paper currency denomination one and 8%, 4%, 2% and 1% in denomination ten, five, fifty and hundred, respectively (Table 3). On the other hand, 10% of the samples were positive for *Salmonella* spp. The prevalence of *Salmonella* spp. was higher in paper currency denomination ten (5%) followed by denomination one (3%). The frequency distribution of *Salmonella* spp. in both denominations five and fifty were equal (1%). However, *Salmonella* spp. was not detected from denomination hundred (Table 3).

**Table 3:** **Prevalence of** ***S. aureus*** **and** ***Salmonella*** **spp. in paper currencies in Jimma town, southwest Ethiopia**

Paper currency denomination	Sample size	No *S. aureus*positive samples (%)	No *Salmonella*spp. positive samples (%)
		Frequency (%)	Frequency (%)
1	20	10 (10)	3 (3.0)
5	20	4 (4.0)	1 (1.0)
10	20	8 (8.0)	5 (5.0)
50	20	3 (2.0)	1 (1.0)
100	20	1 (1.0)	0 (0.0)
Total	100	25 (25.0)	10 (10.0)

Numbers in parenthesis indicate percentage.

### The growth potential of *Salmonella*sp. and *Staphylococcus aureus*in selected weaning foods

**Figure 1:**
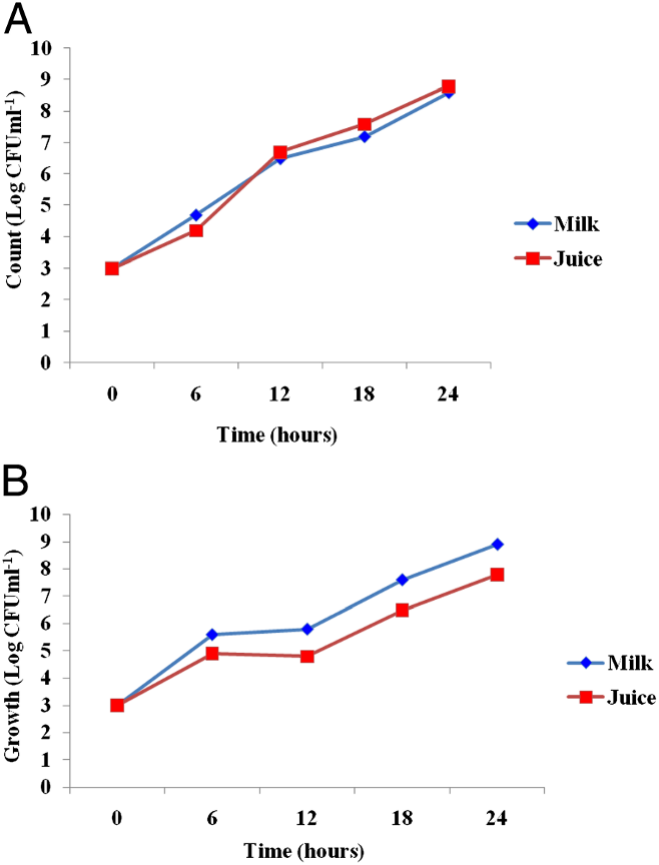
**The growth potential of** ***Salmonella*** **spp (A) and** ***S. aureus*** **(B) isolated from paper currencies in milk and juice, Jimma town, southwestern Ethiopia.**

The count of *Salmonella* sp*.* in each food samples ranged from 2.8 - 3 log CFU ml^-1^at 0 hour (Figure 1). *Salmonella spp*. were observed growing fast in milk (4.7 log CFU ml-1) as compared to its growth in juice (4.2 log CFU ml-1) at 6 hours. The count increased by 1.8 log CFU ml^-1^ and 2.5 log CFU ml^-1^ in milk and juice, respectively, during the first 6 hours (Figure 1). Thereafter, the count reached 6.5 log CFU ml^-1^ in milk and 6.7 log CFU ml^-1^) in juice within 12 hours. The maximum growths attained were 8.6 log CFU ml^-1^ (milk) and 8.8 log CFU ml^-1^ (juice) at 24 hours (Figure 1).

Likewise, the growth potential of *S. aureus* isolated from paper currencies was assessed. Accordingly, higher growth rate was observed in milk (5.6 log CFU ml^-1^) than in juice (4.9 log CFU ml^-1^) at the first 6 hours (Figure 1). The growth rate was slower during the second 6 hours duration as compared to the first 6 hours in juice (4.8 log CFU ml^-1^) and slight increase was observed in milk (5.8 log CFU ml^-1^). The counts increased by 2.2 log in milk (5.8 to 7.6 log CFU ml^-1^) and 1.7 log in juice (4.8 to 6.5 log CFU ml^-1^) at 18 hours. Only slighter growth pattern (1.3 log) was observed in both food samples (7.6 to 8.9 log CFU ml^-1^ in milk and 6.5 to 7.8 log CFU ml^-1^ in juice) at 24 hours (Figure 1).

**Figure 2:**
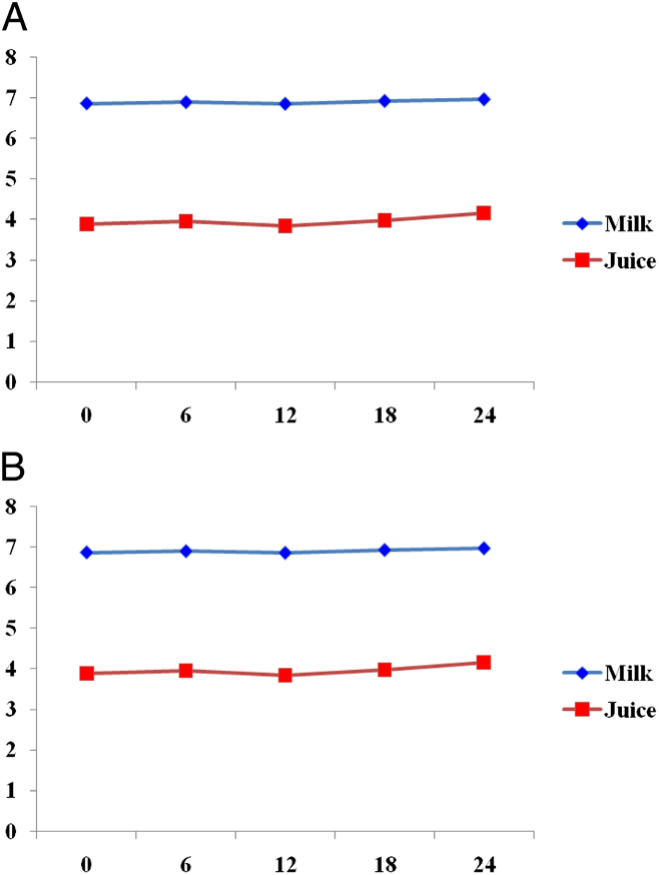
**Change in pH of milk and juice challenged with** ***S. aureus*** **(A) and** ***Salmonella*** **spp. (B) isolated from paper currencies, Jimma town, southwest Ethiopia.**

The pH values of both food samples challenged with *Salmonella* sp*.* were 6.92 (milk) and 3.83 (juice) at 0 hours (Figure 2). There was initial rise in pH from 6.92 to 6.96 (in milk) and 3.83 to 3.98 (in juice) at 6 hours. However, the pH values dropped in both food samples by more than 0.5 (from 6.96 to 6.82 in milk and 3.98 to 3.92 in juice) at 12 hours. Nevertheless, it was raised afterwards up to 24 hours (Figure 2).

The pH values of both food samples challenged by the two pathogens showed some variability in due course of fermentation. However, the overall patterns were gradual increase in pH with drop in degree of acidification mainly due to depletion of carbohydrate sources and reversion towards utilization of proteins and/or release of other metabolites during the 24 hours period (Figure 2).

## Discussion

According to WHO [23], food handling personnel play an important role in ensuring food safety throughout the chain of food production, processing, storage and preparation. Mishandling and disregarding of hygienic measures on the part of food vendors may enable pathogens to get access to food and in some cases to survive and multiply in sufficient numbers to cause illness to the consumers [24]. Consequently, the combination of the widespread use of paper currencies and their constant exchange make them a likely agent for various disease transmissions since communicable diseases can spread through contact with fomites. Besides, a study revealed that paper currencies can serve as an ideal breeding ground for microorganisms [25].

All 100 paper currency samples evaluated in present study were contaminated with different microbial groups. In agreement to several studies [26–32], the predominant bacteria encountered among aerobic mesophilic bacteria in this study were *Staphylococcus* spp. (34.06%) followed by *Bacillus* spp. (31.88%), and Enterobacteraceae (13.39%).

Different studies indicated that the denomination of paper currencies have a direct correlation with degree of contamination as lower denomination notes had the most contaminants [29, 32–34]. Accordingly, the present study showed that lower denomination of Ethiopian paper currencies (1, 5 and 10 notes) had higher degree of contamination with various microorganisms than upper denominations (50 and 100 notes). Thus, from the above fact it can be inferred that lower denomination notes harbor the highest load of microbes, probably because they are exchanged more frequently from one hand to another during market transactions than the higher denomination notes. Moreover, survey conducted elsewhere on mint paper currencies not in circulation were negative for bacterial isolates [13, 35]. In agreement to these report, our study also showed all 20 new paper currency samples not in circulation were negative for bacterial isolates.

Generally, the high mean counts of Enterobacteriaceae on surface swab samples in the present study reveal the poor sanitary condition and poor hygienic practices exercised by those who use the currencies. In addition, coliforms may be introduced to paper currencies by several means; but their presence indicates lack of proper hand washing practice after using toilet. Besides, as Pomperayer and Gaylarde [36] reported, paper currencies are in permanent movement, passing in all environments that constitute a reservoir and source of various bacteria including pathogenic *Escherichia coli*, which can survive 11 days on the inert surfaces of utilities. Therefore, handling of paper currencies may constitute another risk factor for contamination of ready to eat foods unless good hygienic practices are exercised [37].

*Staphylococcus* spp. are found ubiquitously distributed in environment and strains present in the nose often contaminate hands, fingers, faces, and nasal carriers which can easily become skin carriers [38]. Thus, the presence of *Staphylococcus* species on paper currencies could be due to rubbing off or may be surfing from a skin flake [4].

Regarding the prevalence *Staphylococcus aureus,* 25% of the entire paper currency samples in the present study were positive for this bacterium. Our finding is in agreement with earlier reports [4, 29, 39]. In view of the fact that *S. aureus* lives and flourishes in the human nose, throat and skin; the likelihood of frequent recontamination of paper currencies is quite high when good hygienic practices are not in place [40].

In the present study, the prevalence of *Salmonella* spp. was 10%. In other report, too, *Salmonella* spp. were detected in 15% of paper currencies examined from Bangladesh [4]. Moreover, Orukotan and Yabaya [41] reported 4.65% prevalence of *Salmonella* spp*.* in paper currency samples collected from Iran. The detection of *Salmonella* spp. on paper currency could suggest faecal contamination of the paper currencies following poor hygienic practice, potentially resulting in community-acquired infections and disease outbreaks [42]. Moreover, *Salmonella* spp. have marked importance in food borne diseases worldwide [43] besides its capacity to persist on inanimate surfaces for days (up to months) by forming biofilm [44].

The challenge studies showed that the inoculated *Salmonella* spp. reached the level of infective dose (≥5 log CFU ml^-1^) in cow milk and mango juice within 12 hours. The maximum count obtained was 8.6 log CFUml^-1^ (milk) and 8.8 log CFU ml^-1^ (juice) within 24 hours. The observation of the present study was in agreement with the earlier findings of Muleta and Ashenafi [22] where the counts of *Salmonella* spp. were > log 8 CFUg^-1^ within 24 hours in ‘kitfo’ and egg sandwich. On the other hand, Erku and Ashenafi [45] evaluated the growth potential of *Salmonella* spp. in weaning food in Addis Ababa, Ethiopia, where the counts of *Salmonella* reached values as high as log8 CFU ml^-1^ within 12 hours. Thus, based on the current observation, it could be considered that ingestion of foods contaminated with sufficient number of *Salmonella* could induce disease symptoms such as diarrhea, vomiting and fever within the indicated incubation hours [46].

In the present study, the growth potential of *S. aureus* increased considerably in milk with decrease in juice at 12 hours and reached the infective dose within 18 hours in both food samples. The minimum infective dose for *S. aureus* is 6 log CFU g^-1^ [47]. However, Muleta and Ashenafi [22] reported that *S. aureus* reached infective dose in all food samples (macaroni, lentil sandwich and egg sandwich) within 12 hours of initial incubation. In the current study, the maximum count of *S. aureus* recorded within 24 hours was 8.9 log CFU ml^-1^ (milk samples) followed by 7.8 CFUml^-1^ (juice samples). Toxin production is initiated when the *S. aureus* populations exceed 10^6^CFUg^-1^ [48].

In the current study, growth of challenged pathogens generally increased with increase in pH after a period of adaptation and/or modification of the medium. The change in pH with time could be due to the change of source of carbon and nitrogen. Some microbial cultures generate enzymes to utilize a new carbon and energy substrate when a small amount of the original carbon and energy substrate is present [49–51]. However, optimum pH is not the only requirement for the growth of microorganisms as there could be an interplay of both intrinsic and extrinsic parameters that determines the survival and growth of microorganisms in a medium [52].

In general, the current study revealed that paper currencies harbor diverse groups of microorganisms, some of which are potential pathogens. Thus, it calls for awareness development at all levels, especially among personnel working in food establishments, on the possible health risks associated with poor handling of paper currencies while serving/preparing foods. In fact, it should be the concern of all individuals who handle paper money in a circulation directly or indirectly.

Although this study was the first of its kind from Ethiopia, sample collection was delimited to Jimma town. Had the sample collection involved many parts of the country by considering the cultural diversity, hence differences in techniques of food preparation, handling and serving, it would have further strengthened the current findings. Furthermore, the characterization of isolates didn’t involve molecular techniques for much better identification of the same to species and sub-species level. Despite these limitations, the current study generated valuable data to be used for immediate intervention besides serving as a baseline for further study.

## Conclusions

All paper currencies evaluated in this study were contaminated with different microbes including potentially pathogenic *Salmonella* spp. and *S. aureus.* The presence of high number of pathogenic bacteria could cause foodborne diseases of different types including typhoid fever and food poisoning. Moreover, lower denominations which are frequently used by public were more contaminated with various microorganisms than higher denominations. In addition, pathogenic test strains isolated from paper currencies were found growing to infective dose within 12-18 hours indicating that paper currencies are among the risk factors to human health. Thus, periodic evaluation of microbial safety of paper currencies are recommended besides frequent awareness development efforts to improve the existing poor hygienic practices being exercised while handling paper currencies. The government of Ethiopia could also assess the possibility of introducing washable plastic paper currencies to make the cleaning possible without compromising the life spans of paper money in the circulation.
